# Incidence and Survival of Colorectal Cancer in the United Kingdom From 2000 to 2021: A Population-Based Cohort Study

**DOI:** 10.14309/ajg.0000000000003460

**Published:** 2025-04-01

**Authors:** Patricia Pedregal-Pascual, Carlos Guarner-Argente, Eng Hooi Tan, Asieh Golozar, Talita Duarte-Salles, Andreas Weinberger Rosen, Antonella Delmestri, Wai Yi Man, Edward Burn, Daniel Prieto-Alhambra, Danielle Newby

**Affiliations:** 1 Gastroenterology Department, Hospital de la Santa Creu i Sant Pau, Universitat Autònoma de Barcelona, Barcelona, Spain;; 2 Medicine Department, Universitat Autònoma de Barcelona, Barcelona, Spain;; 3 Nuffield Department of Orthopaedics, Rheumatology and Musculoskeletal Sciences, Centre for Statistics in Medicine, University of Oxford, Oxford, UK;; 4 Nemesis Health, New York, USA;; 5 OHDSI Center at the Roux Institute, Northeastern University, Boston, Massachusetts, USA;; 6 Fundació Institut Universitari per a la recerca a l'Atenció Primària de Salut Jordi Gol i Gurina (IDIAPJGol), Barcelona, Spain;; 7 Department of Medical Informatics, Erasmus University Medical Centre, Rotterdam, the Netherlands;; 8 Center for Surgical Science, Zealand University Hospital, Køge, Denmark.

**Keywords:** colorectal cancer, incidence, cancer survival, population-based study

## Abstract

**INTRODUCTION:**

The management of colorectal cancer (CRC) is evolving, with advances in screening and treatment. The purpose of this study was to leverage population-based data to generate up-to-date UK estimates of age-specific and sex-specific incidence and overall survival for the period 2000–2021.

**METHODS:**

We analyzed nationally representative primary care records from Clinical Practice Research Datalink GOLD and replicated in Clinical Practice Research Datalink Aurum. We calculated incidence rates, and short-term and long-term survival stratified by age, sex, and diagnosis year.

**RESULTS:**

Overall incidence was 67.4/100,000 person years, increasing in 2000–2011 to drop slightly in 2011–2014, and then plateauing. By contrast, early-onset CRC raised uninterruptedly throughout the study period, from 8.33 to 19.07/100,000 person-years. Overall survival was 78.3%, 51.4%, and 38.5% at 1, 5, and 10 years, respectively, lower in men compared with women. Modest improvements in survival were observed over the study period, particularly for 60–69-year-old patients.

**DISCUSSION:**

Although the overall incidence in the population has plateaued, a worrying increasing trend of early-onset CRC was observed. Moreover, the slight improvement in overall survival suggests that significant progress is still needed. These findings highlight the urgent need for continued research and resource allocation to improve the diagnosis and management of CRC.

## INTRODUCTION

Colorectal cancers (CRC) are the third most common cancer and the second most common cause of cancer-related death for both men and women worldwide in 2020 (1).

CRC is more common in men, and the risk of developing it increases with advancing age^2^, although there has been a concerning increase in the number of diagnoses among those younger than 50 years of age (2). There are several well-characterized risk factors such as age, family history and hereditary cancer syndromes, inflammatory bowel diseases, metabolic syndrome, sedentary lifestyle, diet, heavy alcohol consumption, and smoking (3). Considering this, there are opportunities for intervention to prevent CRC onset (primary prevention), achieve early detection (secondary prevention), or mitigate its impact on prognosis (tertiary prevention), thereby rendering it a potentially preventable disease (4). CRC screening has been demonstrated to be an effective and cost-effective approach (3). In the United Kingdom, the National Health Service Bowel Cancer Screening Programme began in 2006 (5), is offered every 2 years to those aged 60 to 74, and has now been brought forward to age 50^6^.

Population-based studies using cancer registries have shown an overall increase in incidence in the United Kingdom, but a downward trend has been observed in recent years, especially after 2010, with similar patterns in both sexes (6,7). They also showed an increase in the incidence of CRC in people younger than 50 years (6–8). Furthermore, evidence suggests that CRC mortality in the United Kingdom has declined between 2000 and 2017 (8). However, despite this, the United Kingdom has one of the poorest survival rates compared with the rest of Europe and other high-income countries worldwide (9,10).

Understanding CRC trends is essential for evaluating diagnosis, management, and guiding prevention strategies. Shifts in screening age, lifestyle factors, and rising cases in younger adults have created gaps in trend assessments across UK populations. This study describes the burden of CRC and trends in incidence and survival from 2000 to 2021 using 2 UK primary care databases.

## METHODS

### Study design, setting, and data sources

We performed a population cohort study using primary care data from the United Kingdom. People with a diagnosis of CRC and a denominator population were identified from Clinical Practice Research Datalink (CPRD) GOLD database (July 2022) to estimate overall survival, incidence, and prevalence. We repeated the study using CPRD Aurum (June 2021) to compare the results obtained from GOLD. Both databases contain pseudonymized patient-level information on demographics, lifestyle data, clinical diagnoses, prescriptions, and preventive care provided to patients. CPRD GOLD contains data from across the United Kingdom, whereas Aurum only contains data from England. Both databases were mapped to the Observational Medical Outcomes Partnership Common Data Model (11).

### Study participants and time at risk

All eligible participants were 18 years or older and have at least 1 year of history. History was set to 1 year to allow for characterization of patients before diagnosis and to ensure incidence cases were included for the study. For incidence, the study cohort consisted of individuals present in the database from January 1, 2000. These individuals were followed up to whichever came first: the cancer outcome of interest, exit from the database, date of death, or end of study period (December 31, 2021), for GOLD, and due to data availability of latest extraction, December 31, 2019, for Aurum. For the survival analysis, individuals were followed up from the date of their diagnosis to either date of death, exit from database, or end of the study period.

### Outcome definitions

We used diagnostic codes to identify primary CRC events. Specific diagnostics codes for malignancies of the colon and/or the rectum were included as well as unspecific codes covering these locations because these were assumed to be most likely to be from the colon or rectum. Diagnostic codes indicative of nonmalignant cancer, melanoma, and lymphoma were excluded as well as codes related to cancers of the appendix.

The clinical code list used to define CRC was reviewed by clinicians with oncology, primary care, and real-world evidence expertise (Supplement S1, http://links.lww.com/AJG/D630). For survival analysis, mortality was defined as all-cause mortality based on records of date of death. OMOP-based computable phenotypes are available, together with all analytical code in GitHub to enable research reproducibility (https://github.com/oxford-pharmacoepi/EHDENCancerIncidencePrevalence).

### Statistical methods

The population characteristics of patients with a diagnosis of CRC were summarized, with median and interquartile range used for continuous variables, while counts and percentages were used for categorical variables.

For crude incidence of CRC, number of events, observed time at risk, and the incidence rate (IR) per 100,000 person-years were summarized with 95% CIs. IR was calculated across the entire study period from 2000 to 2021 and annually. Annual IRs were calculated as the number of incident CRC cases as the numerator and the person-years in the general population within that year as the denominator. Using the crude results, age-standardized IRs were calculated using the 2013 European Standard Population (12) and the mid-year population estimates from the office of national statistics 2021 from the United Kingdom (13), with any counts < 5 suppressed to 5 before age standardization. Poisson regression adjusted for year was performed to determine statistical differences between age-standardized IRs between GOLD and Aurum as well as to determine sex differences in IRs within databases. Additional sensitivity analysis included stratification of CRC into colon and rectal cancers, respectively.

For survival analysis, we used the Kaplan-Meier method to estimate the overall survival probability with 95% confidence intervals. We estimated the median survival and survival probability 1, 5, and 10 years after diagnosis. Patients whose death and cancer diagnosis occurred on the same date were removed from the survival analysis.

All results were stratified by age and sex. For survival analysis, we stratified by calendar time of cancer diagnosis. To compare the results with GOLD, the same analysis was performed using CPRD Aurum database, except for stratification by calendar time of cancer diagnosis, which was conducted in GOLD only. Approximate log-rank tests were calculated to assess differences between sex, database, and calendar time. To avoid possible patient identification, we do not report results with fewer than 5 cases.

The statistical software R version 4.2.3 was used for analyses. For calculating incidence and prevalence, we used the IncidencePrevalence R package (14). For survival analysis, we used the survival R package (15).

### Ethics approval and consent to participate

The use of CPRD data for this study was approved by the Independent Scientific Advisory Committee (22_001843). Informed consent was waived by competent authorities because of the anonymized nature of patient data.

## RESULTS

### Patient populations and characteristics

There were 24,340,860 and 11,388,117 eligible people from CPRD Aurum and GOLD, respectively. Attrition tables can be found in the supplementary information (Supplement S2, http://links.lww.com/AJG/D630). A summary of CRC patient characteristics for both databases is given in Table 1. Patients with CRC are more likely to be male (∼56%), with a median age of 72 years old with the highest percentage of cases aged 70–79 years old, contributing to 32% of diagnosed patients.

**Table 1.**
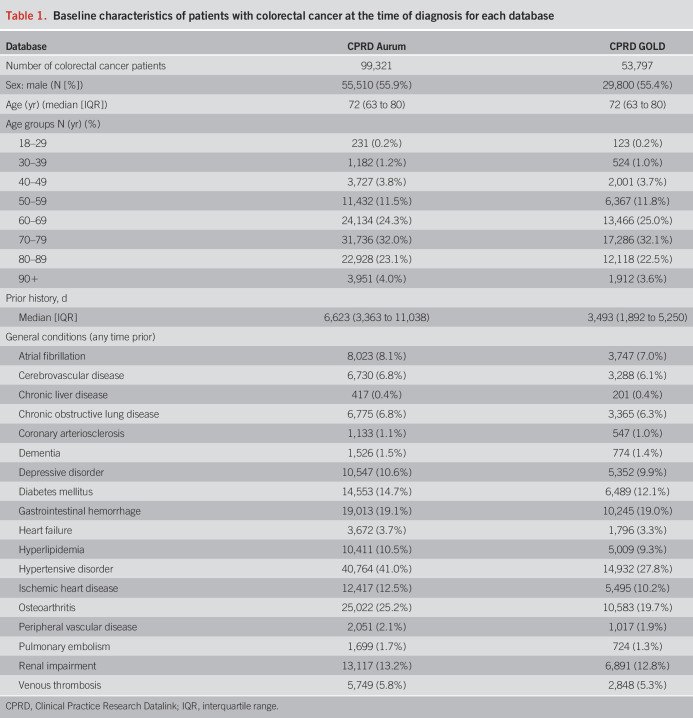
Baseline characteristics of patients with colorectal cancer at the time of diagnosis for each database

### Incidence rates stratified by calendar year, age, and sex

Age-standardized IR across the entire study period from 2000 to 2021 was 67.4 (95% CI 66.9–68.0) per 100,000 person-years for GOLD and 63.7 (95% CI 63.3 to 64.0) for Aurum. For GOLD, sex-specific IRs for men and women were 83.0 (82.0–83.9) and 54.9 (54.2–55.6) per 100,000 person-years, with similar but slightly lower results for Aurum (males 78.3 [77.6 78.9], females 51.5 [51.0 52.0]). All results for this study can be found in an interactive web application: https://dpa-pde-oxford.shinyapps.io/ColorectalCancerIncPrevSurvShiny/.

**Figure 1.**
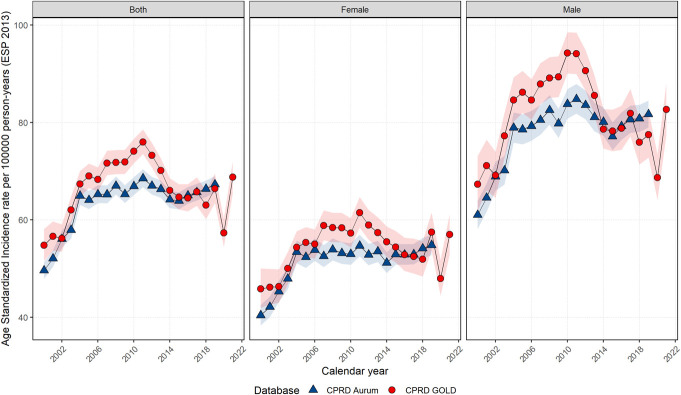
Age-standardized annualized incidence rates for colorectal cancer from 2000 to 2021 (GOLD) and 2000–2019 (Aurum) stratified by database and sex. CPRD, Clinical Practice Research Datalink, ESP 2013, European Standard Population.

Age-standardized yearly IRs increased across the study period for the entire population, with men having higher IRs than women (Figure 1). Poisson regression showed IRs were not statistically different between GOLD and Aurum (*P* value 0.312); however, IRs were statistically significant between sexes within each database (*P* value < 0.001). For men, IRs declined from 2011 to 2014-5 but increased up to 2018 for both databases, increasing in Aurum and decreasing in GOLD. For women, in GOLD, IRs peaked in 2011 before dropping until 2018, while in Aurum, IRs remained stable with a gradual increase toward the end of the study period. For GOLD, there was a drop in IR in 2020 but an increase in IR in 2021. Crude IR trends can be found in the supplement (Supplement S3, http://links.lww.com/AJG/D630). Age-standardized results using mid-year UK population estimates for 2021 showed similar trends (Supplement S4, http://links.lww.com/AJG/D630) and similar trends to national age-standardized cancer statistics (Supplement S5, http://links.lww.com/AJG/D630). Stratification into colon and rectal cancers for CPRD GOLD showed similar IR trends for colon cancer; however, rectal cancer IRs showed a gradual decrease over the study period (Supplement S6, http://links.lww.com/AJG/D630).

IR across the study period (2000–2019/21) increased with age up to 80–89 years. Those aged 18 to 29 had the lowest IRs (0.77 [0.64 to 0.92]) in GOLD, with 80–89 years old having the highest with IRs (277.2 [272.3–282.1]) with similar results in Aurum (Supplement S7, http://links.lww.com/AJG/D630).

**Figure 2.**
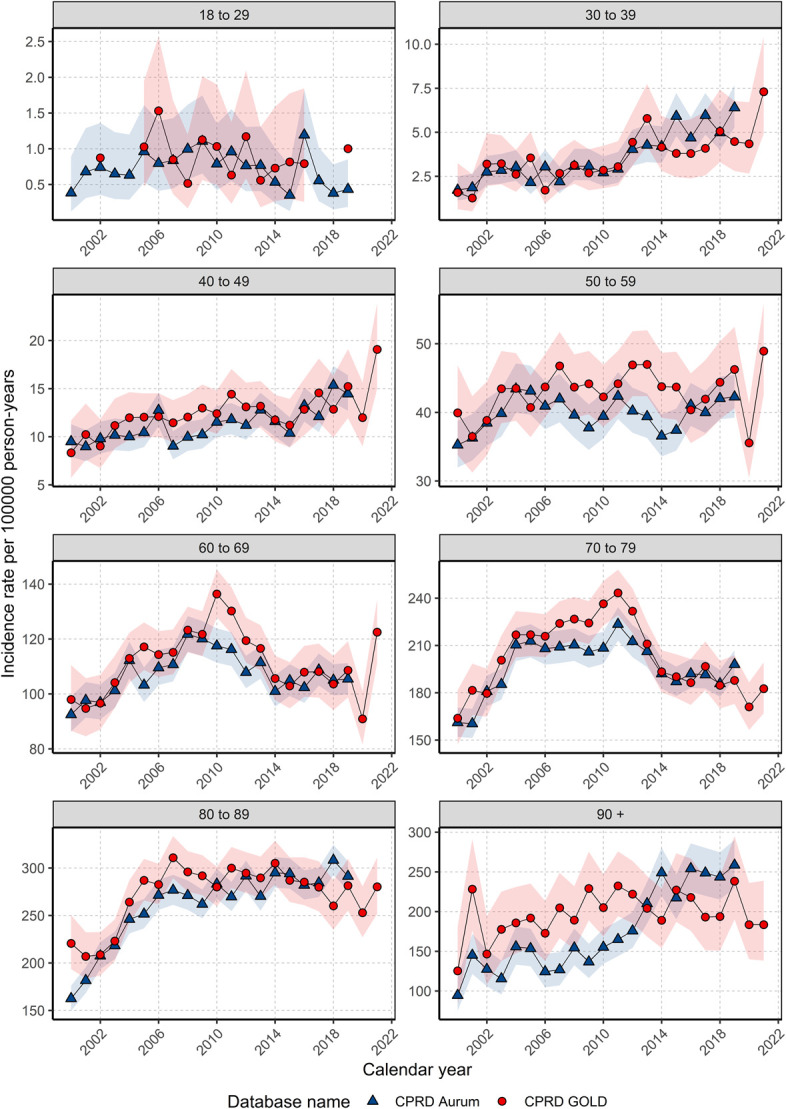
Annualized incidence rates from 2000 to 2021 stratified by database and age group. CPRD, Clinical Practice Research Datalink.

Annualized IRs for each age group (Figure 2) have increased annually in those aged 30 to 59 across both databases. For those aged 60–79 years old, IRs rose and peaked in 2011 before falling and remaining stable. IRs for those aged 80–89 years old remained stable after 2006. For those aged older than 90, IRs in GOLD did not increase from 2011; however in Aurum, IRs increased gradually over the study period. Across most age groups in GOLD, IRs in 2020 decreased before increasing in 2021. Stratification of age groups by 5-year windows showed similar IR trends however with increases only those aged 35 to 44 with a sharp peak in 2021, whereas those aged 45–50 years showed stability for GOLD (Supplement S8, http://links.lww.com/AJG/D630). Trends stratified by both sex and age showed similar age trends in Figure 2 with had higher IRs in men apart from those younger than 50 years of age where there were no sex differences (Supplement S9, http://links.lww.com/AJG/D630). Stratification by site showed similar age trends for colon cancer compared with CRC (Supplement S10, http://links.lww.com/AJG/D630) but for rectal cancer IRs declined over time for those older than 50 with those younger than 50 showing stable IRs (Supplement S11, http://links.lww.com/AJG/D630).

### Overall survival with stratification by sex, age, and calendar year

In GOLD, there were 53,098 patients with 26,084 deaths (49.1%) over the entire study period, whereas for Aurum, there were 98,569 patients with 48,894 deaths (49.6%). Both databases had similar median follow-up time of 2.4–2.5 years and median survival of 5.4–5.5 years (Supplement S12, http://links.lww.com/AJG/D630).

For GOLD, survival after 1, 5, and 10 years from diagnosis was 78.3%, 51.4%, and 38.5%. Women had higher median survival (6.0 [5.8 to 6.3] years) compared with men (5.1 [4.9 to 5.3] years) with similar results obtained in Aurum (males 5.2 years; females 6.13 years). The log-rank test showed no statistically significant differences between survival between databases (*P* value = 0.21). Both sexes had similar 1-year survival (∼79%), whereas men had lower survival at 5 and 10 years than women with survival of 50.3% and 52.8% at 5 years and 36.1% and 40.7% at 10 years for men and women, respectively. Similar results were also obtained in Aurum (Supplement S13, http://links.lww.com/AJG/D630). The log-rank test indicates significant differences between sexes for both databases (*P* value <0.001).

Median survival decreased with increasing age for both databases (Supplement S14, http://links.lww.com/AJG/D630). Survival at 1, 5, and 10 years, decreased from the age of 50 years with the lowest survival observed in those aged >90 years (Supplement S15, http://links.lww.com/AJG/D630). One year survival was between 86% and 90% for those aged 18–49 years compared with those older than 90 years of age where survival was 49%–51%. Five-year survival also decreased from 50 years of age and survival at 10-year decreased from 30 years of age across both databases. Log-rank tests showed significant differences across survival curves (*P* value < 0.001).

**Figure 3.**
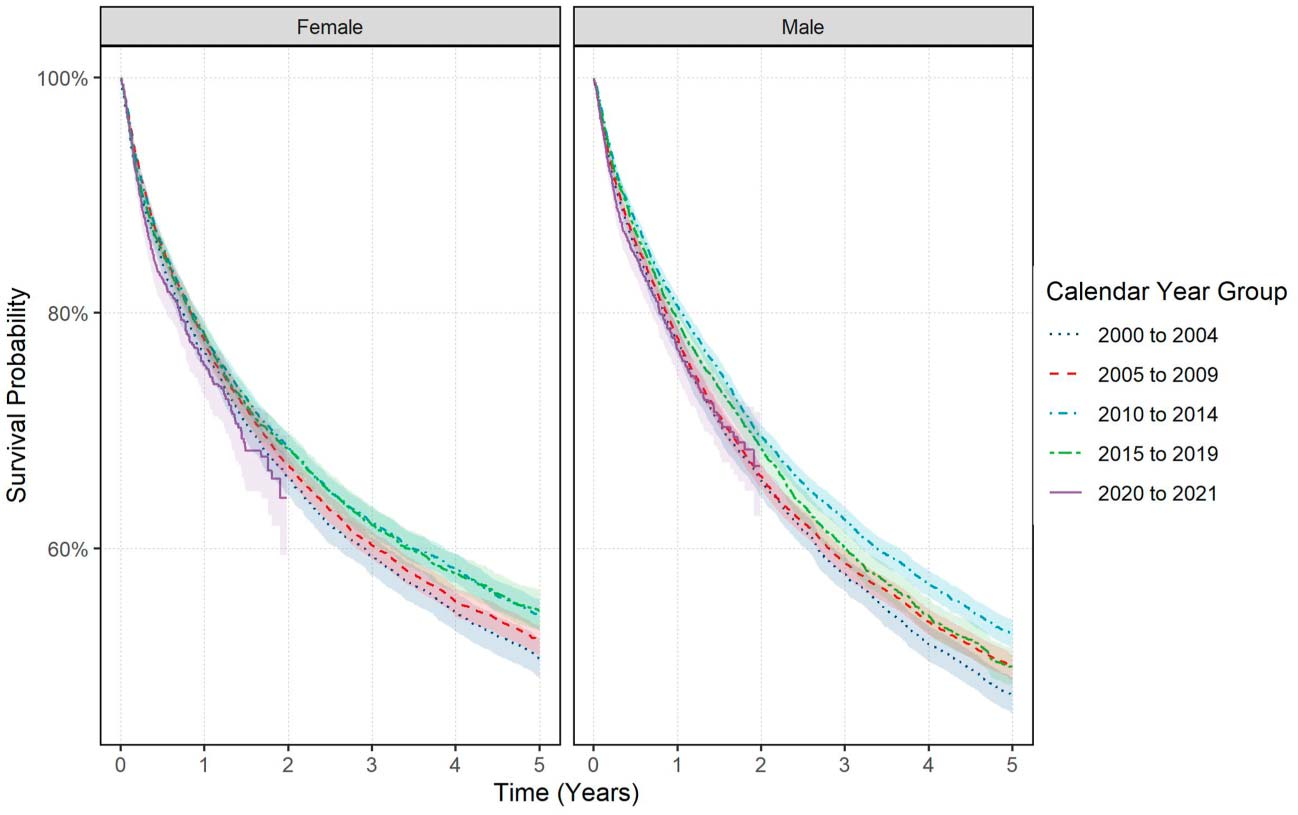
Kaplan-Meier survival curve over 5 years of colorectal cancer stratified by sex and calendar year of diagnosis for Clinical Practice Research Datalink GOLD from 2000 to 2021.

Stratification by calendar time (Figure 3) showed in GOLD, 1- and 5-year survival slightly improved in those diagnosed in 2000 to 2004 to those diagnosed in 2015 to 2019, increasing from 77.1% (76.2–78.0) to 78.9% (78.0–79.67) for 1-year survival and from 48.9% (47.8–50.0) to 52.0% (50.9–53.2) for 5-year survival with the log-rank test also showing statistical significance with calendar year groups (*P* < 0.001). Stratification by sex showed improvements in 5-year survival for both sexes, but not for 1-year survival with log-rank tests showing statistical significance for each sex (*P* < 0.001) (Supplement S16, http://links.lww.com/AJG/D630). Stratification by age showed only those aged 60–69 years showed improvements in 1-year (80.7% [79.0–82.5] to 85.8% [84.4–97.2]) and 5-year survival (55.5% [53.3–57.8] to 64.1% [61.9–66.4]) comparing those diagnosed in 2000–2004 with those diagnosed in 2015–2019 with similar results for both sexes. Log-rank tests showed statistical significance (<0.001) apart from those aged 18 to 39 and older than 90 years of age. There was no difference in survival for those diagnosed in 2020–2021 to previous calendar year groups.

## DISCUSSION

This study provides a comprehensive study of the trends of CRC incidence and survival in the United Kingdom. Incidence increased until 2011 with a relevant increase in those younger than 50 years of age. Survival slightly improved from 2000 to 2019 particularly in those aged 60–69 years.

Our estimates and trends of IRs are in line with national statistics (16). Similar to previous literature, we also observed higher rates in men and older people (3,17,18).

We observed 4 main trends on incidence during the study period. First, a progressive increase after 2006, reaching its peak in 2011 and decreasing the subsequent years, with a sharp rise in 2021, likely driven by deferred diagnosis after the pandemic (19). Earlier trends could be associated with the introduction of the Screening Programme (20), with increased IRs from detecting asymptomatic cases followed by a reduction due to endoscopic adenoma resection (4,21). Other studies showed the same pattern (7,8). For example, a population-based study in the Netherlands reported an initial rise in early-stage CRC incidence after screening began, followed by a decline in both early-stage and advanced-stage cases (22). Furthermore, these patterns seem to be driven by colon cancer rather than rectal cancer which has been declined potentially due to a combination of screening, lifestyle shifts, and different risk factors. Age-specific IRs may not strongly reflect the impact of population screening programmes in the short term. This is shown in this study where we did not see a decrease in incidence in those aged 60–69 years from 2006. However, although the screening programme began in 2006, it was not fully implemented until 2009; therefore, the effects of screening may not be apparent after 5–10 years. Moreover, low participation rates could also attenuate the impact of population screening on incidence (23).

Third, we observed a worrying increase in cases in those younger than 50 years of age, like in many other countries (2,7). The reasons for this increase are unclear. Early-onset CRC (EOCRC) seems to have differential clinical and pathological characteristics (24). One possible explanation is birth cohort effects (25,26), where those born after 1960s were exposed to modifiable risk factors such as unhealthy Western diets, sedentary behavior, and lower physical activity (25,27). In fact, this increase seems to be driven by those aged 35–44 years, as we observe stability in those aged 45–50 years, supporting the theory of birth-cohort effects and probably increased awareness of symptoms. Interestingly, we did not observe sex differences in this age group, suggesting that these exposures are not sex-specific (2,28,29). On the other hand, the rising incidence of CRC in younger age groups has led to some countries, including the United Kingdom, to lower the bowel cancer screening age eligibility (30). Notably, Austria and Italy, with lower screening eligibility, show a decline in young-onset CRC, while incidence rises in 9 other high-income countries (26). However, although incidence increased in younger individuals, the absolute risk in this age group is much lower compared with those aged older than 80, whose incidence remained stable. This difference in risk magnitude highlights the greater burden of the disease in older populations and should be considered when evaluating overall trends.

Fourth, the IRs also increased over the study period in nonagenarians. This could be due to improvements in life expectancy (31). In addition, this age group does not benefit of routine cancer screening because it is not recommended older than 85 years of age (31). Diagnosing and treating this age group is challenging because colonoscopy poses risks and may yield incomplete examinations in elderly or frail patients (32). Several studies suggest that age should not be a relevant criterion; instead, older patients should be included in clinical trials and receive geriatric assessment to optimize cancer care (29,33). However, a population-based study found age remains a major factor in UK treatment decisions (34).

Like other studies (3,18,35,36), we observed higher IRs and worse overall survival in man, except for those with EOCRC. Although negligible differences in age-standardized survival among men and women had been reported in England (18), other studies found that women have better overall, cancer-specific, and recurrence-free survival than men (37). Sex differences can be attributed to various modifiable and nonmodifiable factors. Men face greater exposure and vulnerability to environmental risk factors (3,36,38), and have lower CRC screening uptake, although a higher proportion of CRCs in men are diagnosed through screening in England (18). This may be due to women being more likely to have proximal and aggressive cancers, as well as the lower effectiveness of guaiac Fecal Occult Blood Test/Fecal Immunochemical Test screening in women (18). In addition, sexual dimorphisms may also play a role in these differences (36).

Short-term and long-term survival results are in line with national statistics (39) and with previous literature (9). Median survival decreased with age as expected, which can be explained by frailty and comorbidities (40). Despite improvements in therapeutic management, survival impact has been modest, with only those aged 60–69 showing consistent improvements, likely due to screening effects in this group (41). Compared with other European countries with similar health systems and populations, overall survival rates are worse in the United Kingdom, particularly for elderly people (9,10). Careful consideration is necessary when interpreting these comparisons because these studies are based on cancer registries and there are intercountry differences in data collection, potentially introducing bias (42). Efforts are increasing to understand CRC's extensive heterogeneity to move beyond the current “one-size-fits-all” therapeutic strategy. Emerging research is focusing on personalized treatments based on tumor molecular characteristics, improving risk stratification, chemotherapy monitoring, and early relapse detection (43,44). Once applied in clinical practice, these advancements are expected to enhance survival rates in the coming years.

This study has many strengths. First, we used 2 large primary care databases covering a large proportion of the United Kingdom (45,46), and the consistency between them supports the robustness of our findings. The sharp increase in incidence from 2000 to 2004 may reflect the introduction of the Quality and Outcomes Framework in 2004, which encouraged general practitioners to record all new cases (47). In addition, our representative population-based databases allowed for direct assessments of incidence and prevalence, while cancer registry studies typically use extrapolations to estimate data for the broader population, which could potentially introduce bias (48,49). Finally, the high validity of mortality data (98% accuracy) (50) allowed us to examine the impact of calendar time on overall survival.

One limitation is the lack of linkage to cancer registry data, which may result in misclassification or delayed diagnoses. However, previous studies have shown that cancer diagnoses in UK primary care records are highly accurate and complete (51). Second, our use of primary care records precluded us from stratification by tumor histology, genetic mutations, staging, or cancer therapies, so we were unable to account for differences in prognosis across subgroups. This limitation may affect survival estimates, particularly for those with advanced-stage disease (41) or high-risk genetic mutations (52,53). Furthermore, staging information from the national cancer registry (England only) is very incomplete before pre-2012 (51) with linkage only available up to 2018 which only covered part of our study period which would give a poor estimate of survival particularly with the implementation and changes to population screening. Factors such as socioeconomic status and ethnicity may also affect incidence, prevalence, and survival (54). We only excluded individuals with the same date of death and cancer diagnosis, which may have led to an underestimation of survival times if there was a lag between diagnosis and death that was not captured. Finally, the use of overall survival rather than net survival may mean comparisons across populations with varying comorbidity burdens may need careful interpretation. However, overall survival is a valid and useful measure routinely used in observational studies and clinical trials reflecting real-world outcomes by accounting for both cancer-related and non-cancer-related deaths. This makes it particularly relevant in older populations, where comorbidities and competing risks affect survival estimates.

In conclusion, our study shows a reduction in CRC incidence over the years, mostly driven by those aged 60–79 years, in whom consistent small survival improvements are observed, possibly associated with screening. The rising EOCRC incidence raises the question of whether lowering the screening age could improve outcomes and highlights the need to better understand its biology and risk factors for more targeted screening. Finally, despite therapeutic advancements, we found only modest survival improvements, likely due to multiple factors, underscoring the need for further research into CRC management.

## CONFLICTS OF INTEREST

**Guarantor of the article:** Danielle Newby, PhD.

**Specific author contributions:** All authors were involved in the study conception and design, interpretation of the results, and the preparation of the manuscript. A.D. and W.Y.M.: implemented the data curation, data harmonisation, data quality tests and assessment. D.N.: carried out data analysis for the manuscript. A.W.R.: developed the clinical code lists for CRC for this study. A.G. and A.W.R.: reviewed the clinical codelists used in this study. P.P.P., C.G.A., and D.N.: wrote the initial draft of the manuscript with D.P.A., D.N., E.B., A.D., W.Y.M., and D.P.A.: had access to the CPRD data. All authors critically reviewed the final manuscript and gave consent for publication.

**Financial support:** This activity under the European Health Data & Evidence Network (EHDEN) has received funding from the Innovative Medicines Initiative 2 (IMI2) Joint Undertaking under grant agreement No 806968. IMI2 receives support from the European Union's Horizon 2020 research and innovation programme and European Federation of Pharmaceutical Industries and Associations (EFPIA). The sponsors of the study did not have any involvement in the writing of the manuscript or the decision to submit it for publication. Additionally, there was partial support from the Oxford NIHR Biomedical Research Centre. The corresponding author had full access to all the data in the study and had final responsibility for the decision to submit for publication.

**Potential competing interests:** Professor Daniel Prieto-Alhambra's research group from the University of Oxford has received research grants from the European Medicines Agency, from the Innovative Medicines Initiative, from Gilead Science, and from UCB Biopharma. EHT received consultancy fees from Janssen Pharmaceutica NV, outside the submitted work. All other authors declare no conflicts of interest.

**Data availability:** This study is based in part on data from the Clinical Practice Research Datalink (CPRD) obtained under licence from the UK Medicines and Healthcare Products Regulatory Agency. The data are provided by patients and collected by the NHS as part of their care and support. The interpretation and conclusions contained in this study are those of the author/s alone. Patient-level data used in this study were obtained through an approved application to the CPRD (application number 22_001843) and are only available following an approval process to safeguard the confidentiality of patient data. Details on how to apply for data access can be found at https://cprd.com/data-access

**Study Highlights**

**WHAT IS KNOWN**

✓ A stabilization or decline in colorectal cancer (CRC) incidence is observed in the past few decades in most developed countries.
✓ A concerning increase in the diagnosis of CRC in young patients (early-onset CRC) is also observed.

**WHAT IS NEW HERE**

✓ This is the first study conducted using real-world, population-level data with accurate real-time disease-free denominators.
✓ Overall incidence of CRC has stabilized, but early-onset CRC raised uninterruptedly throughout the study period.
✓ Modest improvements in survival were observed over the study period.

## Supplementary Material
