# Supplementary material for: Incidence and Survival of Colorectal Cancer in the United Kingdom From 2000 to 2021: A Population-Based Cohort Study
Source: Am J Gastroenterol. 2025 Apr 1;120(12):2909–17. doi: 10.14309/ajg.0000000000003460 (PMC12662139; doi:10.14309/ajg.0000000000003460)
Supplement: Supplementary file 1 [file acg-120-2909-s001.docx]

**Supplementary Information**

Supplemental to incidence, prevalence, and survival of colorectal cancer in the United Kingdom from 2000-2021: a population-based cohort study

[**S1: Clinical codelists for colorectal cancer**  2](#_Toc189587388)

[**S2: Population attrition showing eligible patients for study from each database.** 4](#_Toc189587389)

[**S3: Crude annual incidence rates for CPRD GOLD and CPRD Aurum for CRC stratified by sex.** 5](#_Toc189587390)

[**S4: Age Standardized annual incidence rates for CPRD GOLD and CPRD Aurum for CRC stratified by sex using mid-year population estimates from the UK 2021.** 5](#_Toc189587391)

[**S5: Comparison of age standardized annualized incidence rates of CRC from this study with National Cancer Registration and Analysis Service (NCRAS) rates.** 6](#_Toc189587392)

[**S6: Age Standardized annual incidence rates for CPRD GOLD for colon and rectal cancer stratified by sex using European Standard Population 2013.** 6](#_Toc189587393)

[**S7: Crude Incidence rates for whole study period (2000-2021 GOLD, 2000-2019 Aurum) for CRC from stratified by database and age group.** 7](#_Toc189587394)

[**S8: Annualised incidence rates for CRC for CPRD GOLD stratified by database, sex, and age group in 5-year age bands.** 8](#_Toc189587395)

[**S9: Annualised incidence rates for colorectal cancer stratified by database, sex, and age group.** 9](#_Toc189587396)

[**S10: Age Standardized annual incidence rates for colon cancer stratified by age group for CPRD GOLD.** 10](#_Toc189587397)

[**S11: Age Standardized annual incidence rates for rectal cancer stratified by age group for CPRD GOLD.** 11](#_Toc189587398)

[**S12: Kaplan-Meier survival curve of colorectal cancer by database and sex** 12](#_Toc189587399)

[**S13: Survival (%) after 1, 5 and 10 years after colorectal cancer diagnosis stratified by database and sex.** 12](#_Toc189587400)

[**S14: Median survival stratified by database and age group.** 13](#_Toc189587401)

[**S15: One-, five- and ten-year survival (95% confidence intervals) of colorectal cancer stratified by database and age group.** 14](#_Toc189587402)

[**S16: Survival after 1 and 5 years stratified by database, calendar year for whole population and sex.** 15](#_Toc189587403)

# **S1: Clinical codelists for colorectal cancer**

The clinical codelists used for colorectal cancer is listed in the table below with the corresponding SNOMED concept ID, OMOP concept ID and concept description. Only diagnosis records alone were used to identify cancer outcome for this study. We developed concept definitions using ATLAS, the OHDSI open-source platform (<https://github.com/OHDSI/atlas>). Clinical adjudicators reviewed the cohort definitions and associated concept sets.

| **Concept Id** | **Concept SNOMED Code** | **Concept Description** |
| --- | --- | --- |
| 4180790 | 363406005 | Malignant tumor of colon |
| 443390 | 363351006 | Malignant tumor of rectum |
| 4110575 | 254582000 | Adenocarcinoma of rectum |
| 443381 | 363410008 | Malignant tumor of sigmoid colon |
| 443391 | 363350007 | Malignant tumor of cecum |
| 435754 | 363412000 | Malignant tumor of ascending colon |
| 4180792 | 363414004 | Malignant tumor of rectosigmoid junction |
| 443384 | 363408006 | Malignant tumor of transverse colon |
| 4116240 | 255081007 | Carcinoma of cecum |
| 4180791 | 363407001 | Malignant tumor of hepatic flexure |
| 443382 | 363409003 | Malignant tumor of descending colon |
| 4181344 | 363413005 | Malignant tumor of splenic flexure |
| 4198567 | 315058005 | HNPCC - hereditary nonpolyposis colon cancer |
| 4089661 | 187757001 | Malignant neoplasm, overlapping lesion of colon |
| 74582 | 93984006 | Primary malignant neoplasm of rectum |
| 79740 | 109838007 | Overlapping malignant neoplasm of colon |
| 197500 | 93761005 | Primary malignant neoplasm of colon |
| 432257 | 94105000 | Primary malignant neoplasm of transverse colon |
| 432837 | 371977004 | Primary malignant neoplasm of cecum |
| 436635 | 94006002 | Primary malignant neoplasm of sigmoid colon |
| 437798 | 94072004 | Primary malignant neoplasm of splenic flexure of colon |
| 438090 | 109839004 | Overlapping malignant neoplasm of rectum, anus and anal canal |
| 438699 | 93980002 | Primary malignant neoplasm of rectosigmoid junction |
| 438979 | 93826009 | Primary malignant neoplasm of hepatic flexure of colon |
| 441800 | 93771007 | Primary malignant neoplasm of descending colon |
| 443396 | 363510005 | Malignant tumor of large intestine |
| 764981 | 98981000119103 | Primary malignant neoplasm of ileocecal valve |
| 4115028 | 285312008 | Carcinoma of sigmoid colon |
| 4149847 | 269533000 | Carcinoma of colon |
| 4151260 | 269544008 | Carcinoma of the rectosigmoid junction |
| 4184850 | 413446001 | Adenocarcinoma of cecum |
| 4193165 | 312113007 | Carcinoma of descending colon |
| 4193871 | 312112002 | Carcinoma of transverse colon |
| 4193872 | 312115000 | Carcinoma of splenic flexure |
| 4200514 | 301756000 | Adenocarcinoma of sigmoid colon |
| 4207182 | 312111009 | Carcinoma of ascending colon |
| 4207183 | 312114001 | Carcinoma of hepatic flexure |
| 4246125 | 93854002 | Primary malignant neoplasm of large intestine |
| 4247719 | 93683002 | Primary malignant neoplasm of ascending colon |
| 4256776 | 408645001 | Adenocarcinoma of large intestine |
| 4307687 | 422581008 | Carcinoma of colon, stage II |
| 4310858 | 422375001 | Carcinoma of colon, stage III |
| 4312001 | 422985007 | Carcinoma of colon, stage IV |
| 4312240 | 425213009 | Carcinoma of colon, stage I |
| 4322376 | 425178004 | Adenocarcinoma of rectosigmoid junction |
| 36683531 | 781382000 | Malignant neoplasm of colon and/or rectum |
| 36713361 | 681601000119101 | Primary adenocarcinoma of ascending colon |
| 36715911 | 721695008 | Primary adenocarcinoma of ascending colon and right flexure |
| 36715912 | 721696009 | Primary adenocarcinoma of transverse colon |
| 36717495 | 721699002 | Primary adenocarcinoma of descending colon and splenic flexure |
| 37016239 | 184881000119106 | Primary adenocarcinoma of rectosigmoid junction |
| 37018659 | 96281000119107 | Overlapping malignant neoplasm of colon and rectum |
| 37208245 | 681651000119102 | Primary adenocarcinoma of descending colon |
| 40492939 | 448994001 | Carcinoma of upper rectum |
| 42537577 | 737058005 | Microsatellite instability-high colorectal cancer |
| 42872396 | 1701000119104 | Primary adenocarcinoma of colon |

# **S2: Population attrition showing eligible patients for study from each database.**

| **N** | **Reason** | **N excluded** | **Database** |
| --- | --- | --- | --- |
| 39999011 | Starting population |  | Aurum |
| 39999011 | Missing year of birth | 0 |  |
| 39999011 | Missing sex | 0 |  |
| 34833388 | Cannot satisfy age criteria during the study period based on year of birth | 5165623 |  |
| 29190480 | No observation time available during study period | 5642908 |  |
| 29190480 | Doesn't satisfy age criteria during the study period | 0 |  |
| 25483313 | Prior history requirement not fulfilled during study period | 3707167 |  |
| 24340860 | No observation time available after applying age and prior history criteria | 1142453 |  |
| 24340860 | Starting analysis population |  |  |
| 24340860 | Estimating prevalence |  |  |
| 24319115 | Excluded due to prior event (do not pass outcome washout during study period) | 21745 |  |
| 24319115 | Estimating incidence |  |  |
| 99321 | With a cancer diagnosis | 24219794 |  |
| 98569 | Cancer diagnosis not on same date as death | 752 |  |
| 86710 | Estimating survival |  |  |
| 17054819 | Starting population |  | GOLD |
| 17054819 | Missing year of birth | 0 |  |
| 17054819 | Missing sex | 0 |  |
| 15210165 | Cannot satisfy age criteria during the study period based on year of birth | 1844654 |  |
| 13978229 | No observation time available during study period | 1231936 |  |
| 13978229 | Doesn't satisfy age criteria during the study period | 0 |  |
| 12254874 | Prior history requirement not fulfilled during study period | 1723355 |  |
| 11388117 | No observation time available after applying age and prior history criteria | 866757 |  |
| 11388117 | Starting analysis population |  |  |
| 11388117 | Estimating prevalence |  |  |
| 11381775 | Excluded due to prior event (do not pass outcome washout during study period) | 6342 |  |
| 11381775 | Estimating incidence |  |  |
| 53797 | With a cancer diagnosis | 11327978 |  |
| 53098 | Cancer diagnosis not on same date as death | 699 |  |
| 53098 | Estimating survival |  |  |

# **S3: Crude annual incidence rates for CPRD GOLD and CPRD Aurum for CRC stratified by sex.**


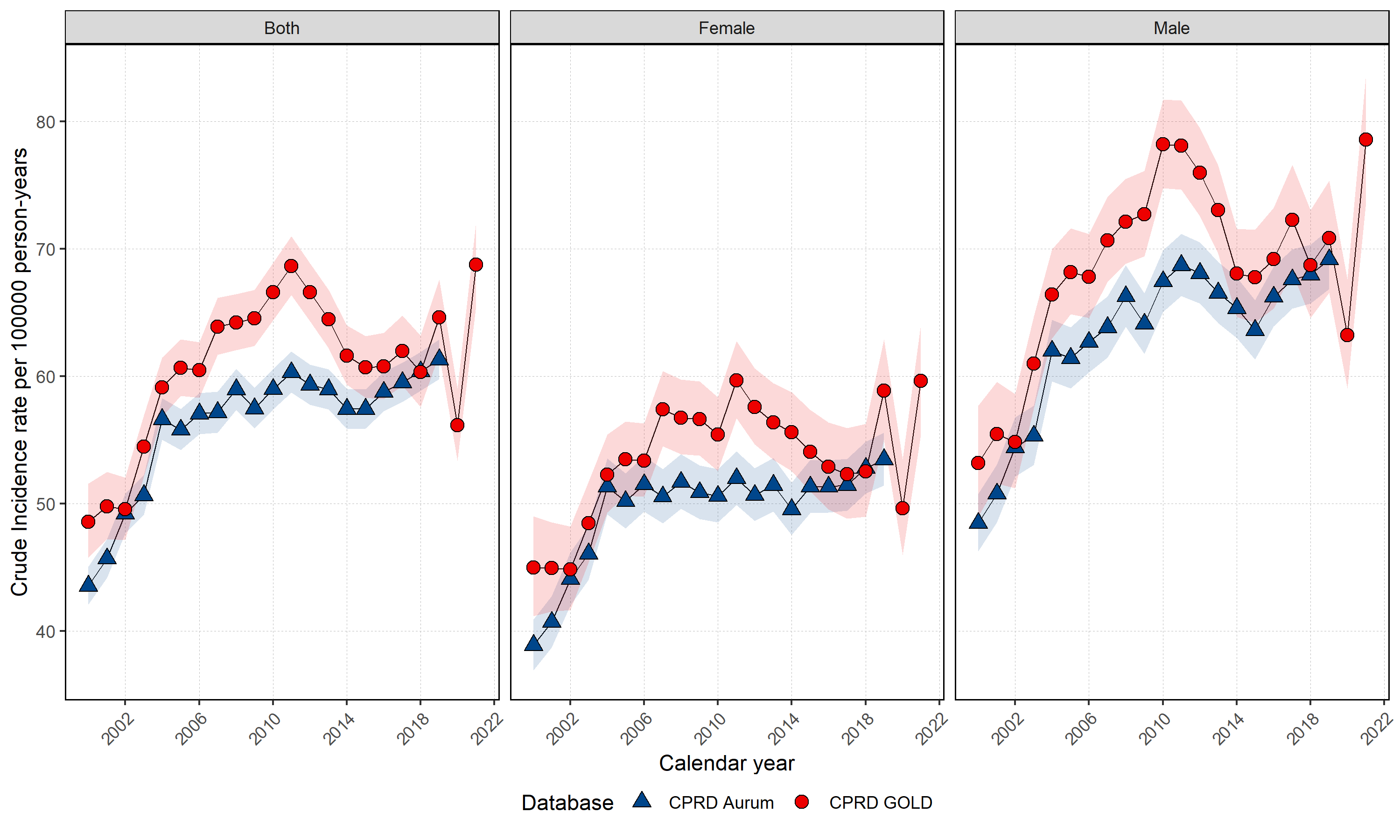


# **S4: Age Standardized annual incidence rates for CPRD GOLD and CPRD Aurum for CRC stratified by sex using mid-year population estimates from the UK 2021.**


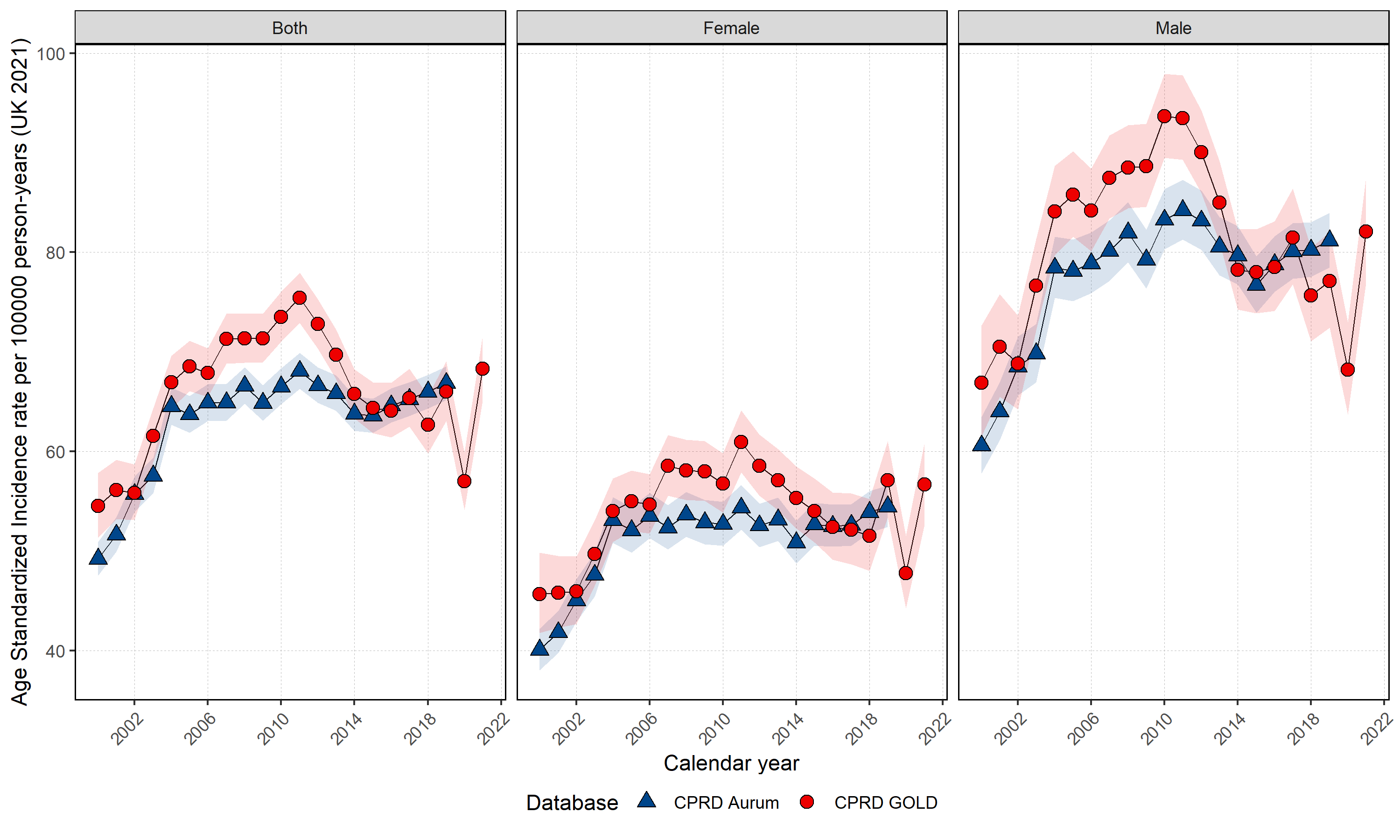


# **S5: Comparison of age standardized annualized incidence rates of CRC from this study with National Cancer Registration and Analysis Service (NCRAS) rates.**

**
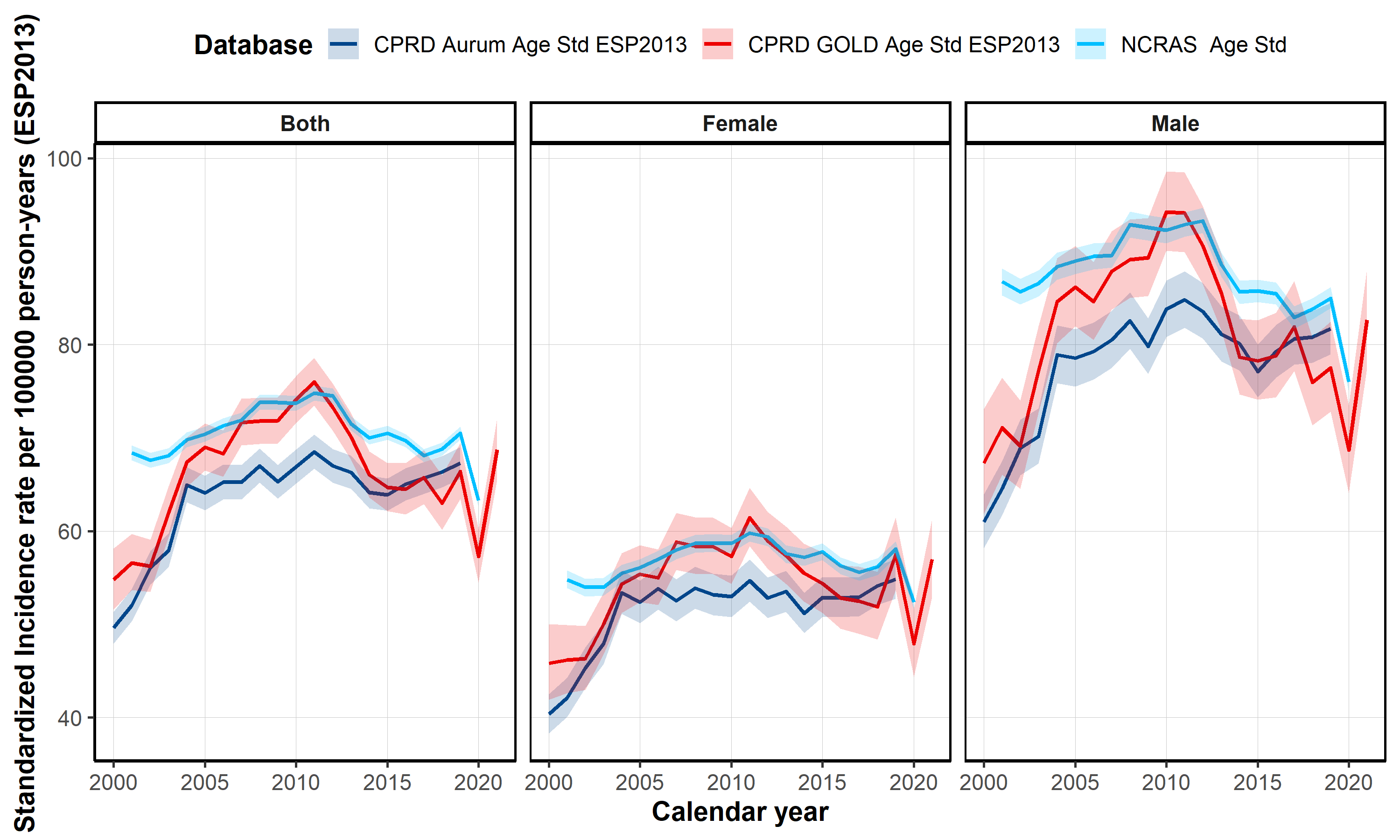
**

# **S6: Age Standardized annual incidence rates for CPRD GOLD for colon and rectal cancer stratified by sex using European Standard Population 2013.**


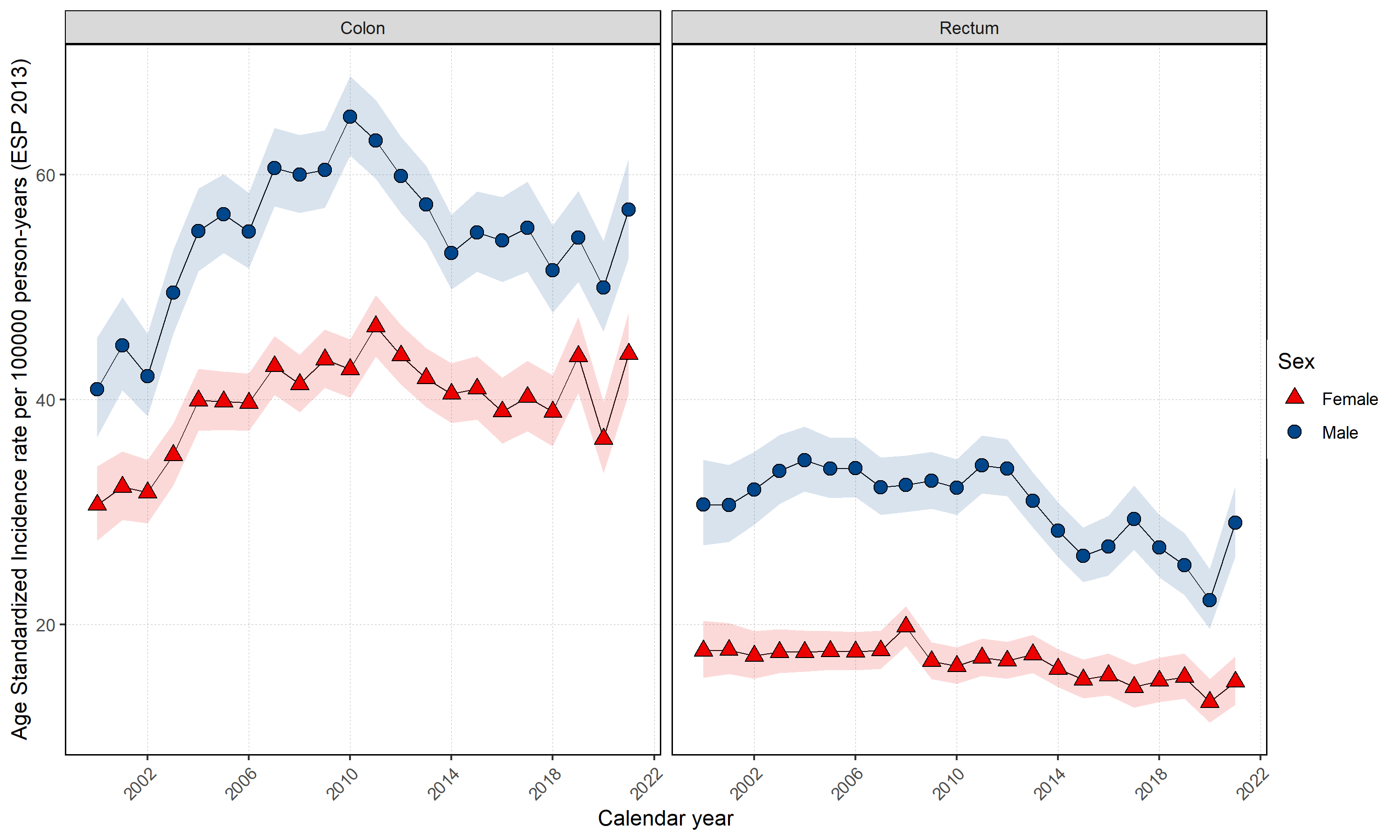


# **S7: Crude Incidence rates for whole study period (2000-2021 GOLD, 2000-2019 Aurum) for CRC from stratified by database and age group.**

| **Age Group** | **n persons** | **person years** | **n events** | **Incidence (100000 pys)** | **Database** |
| --- | --- | --- | --- | --- | --- |
| 18 to 29 | 9,238,374 | 31,941,017 | 231 | 0.72 (0.63 to 0.82) | CPRD Aurum |
| 30 to 39 | 8,292,235 | 32,676,728 | 1,182 | 3.62 (3.41 to 3.83) |  |
| 40 to 49 | 6,514,825 | 32,912,807 | 3,727 | 11.32 (10.96 to 11.69) |  |
| 50 to 59 | 5,434,527 | 28,632,117 | 11,432 | 39.93 (39.20 to 40.67) |  |
| 60 to 69 | 4,161,289 | 22,412,580 | 24,134 | 107.68 (106.33 to 109.05) |  |
| 70 to 79 | 3,102,304 | 16,108,479 | 31,736 | 197.01 (194.85 to 199.19) |  |
| 80 to 89 | 1,930,511 | 8,707,470 | 22,928 | 263.31 (259.92 to 266.74) |  |
| 90 + | 658,438 | 2,239,680 | 3,951 | 176.41 (170.95 to 182.00) |  |
| 18 to 29 | 3,871,109 | 16,018,361 | 123 | 0.77 (0.64 to 0.92) | CPRD GOLD |
| 30 to 39 | 3,682,270 | 15,106,025 | 524 | 3.47 (3.18 to 3.78) |  |
| 40 to 49 | 3,246,464 | 16,111,285 | 2,001 | 12.42 (11.88 to 12.98) |  |
| 50 to 59 | 2,884,106 | 14,703,972 | 6,367 | 43.30 (42.24 to 44.38) |  |
| 60 to 69 | 2,289,865 | 11,852,052 | 13,466 | 113.62 (111.71 to 115.55) |  |
| 70 to 79 | 1,675,784 | 8,346,824 | 17,286 | 207.10 (204.02 to 210.21) |  |
| 80 to 89 | 1,016,741 | 4,372,137 | 12,118 | 277.16 (272.25 to 282.14) |  |
| 90 + | 319,907 | 955,339 | 1,913 | 200.24 (191.37 to 209.42) |  |

**Pys: person years**

# **S8: Annualised incidence rates for CRC for CPRD GOLD stratified by database, sex, and age group in 5-year age bands.**


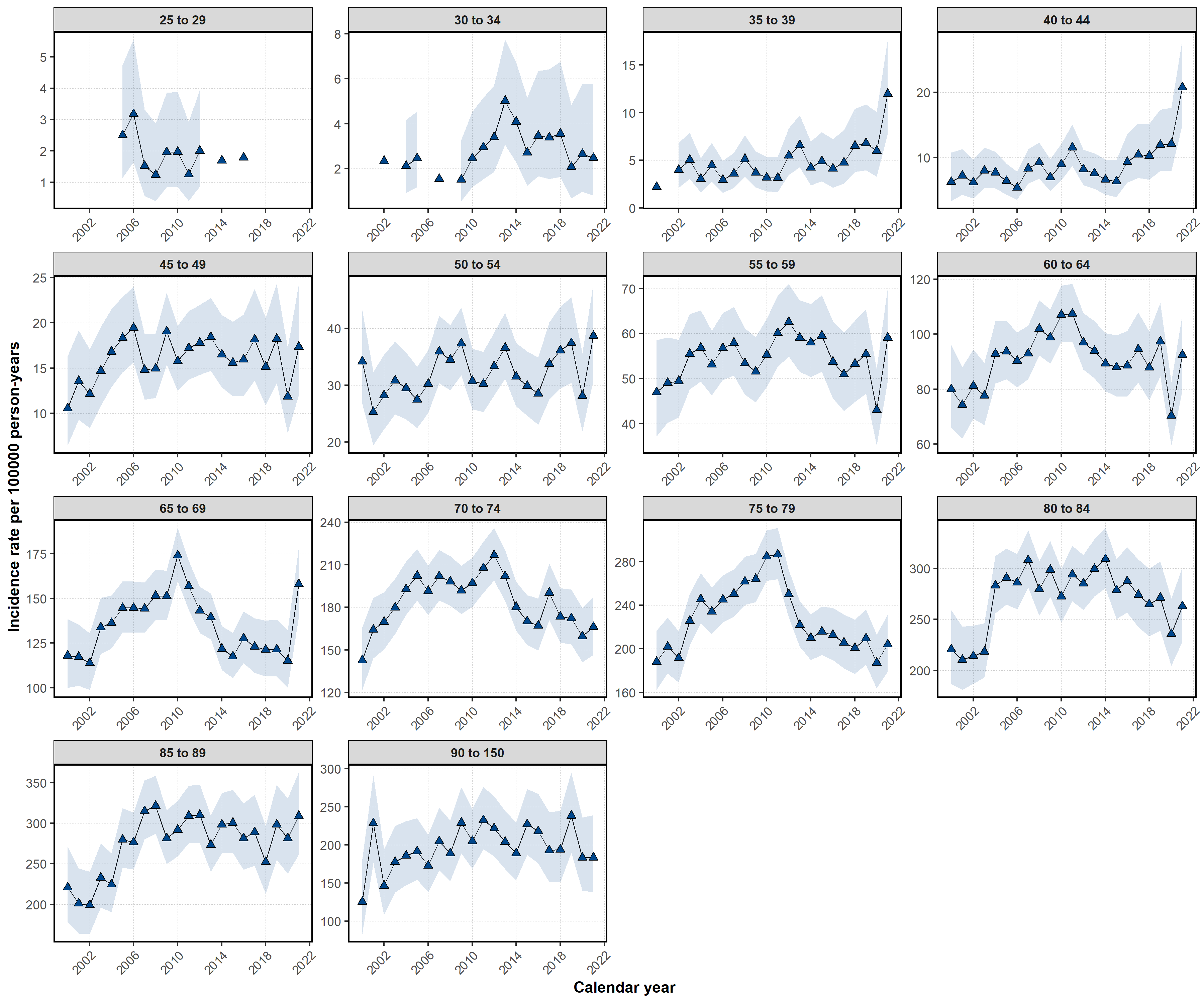


# **S9: Annualised incidence rates for colorectal cancer stratified by database, sex, and age group.**





# **S10: Age Standardized annual incidence rates for colon cancer stratified by age group for CPRD GOLD.**


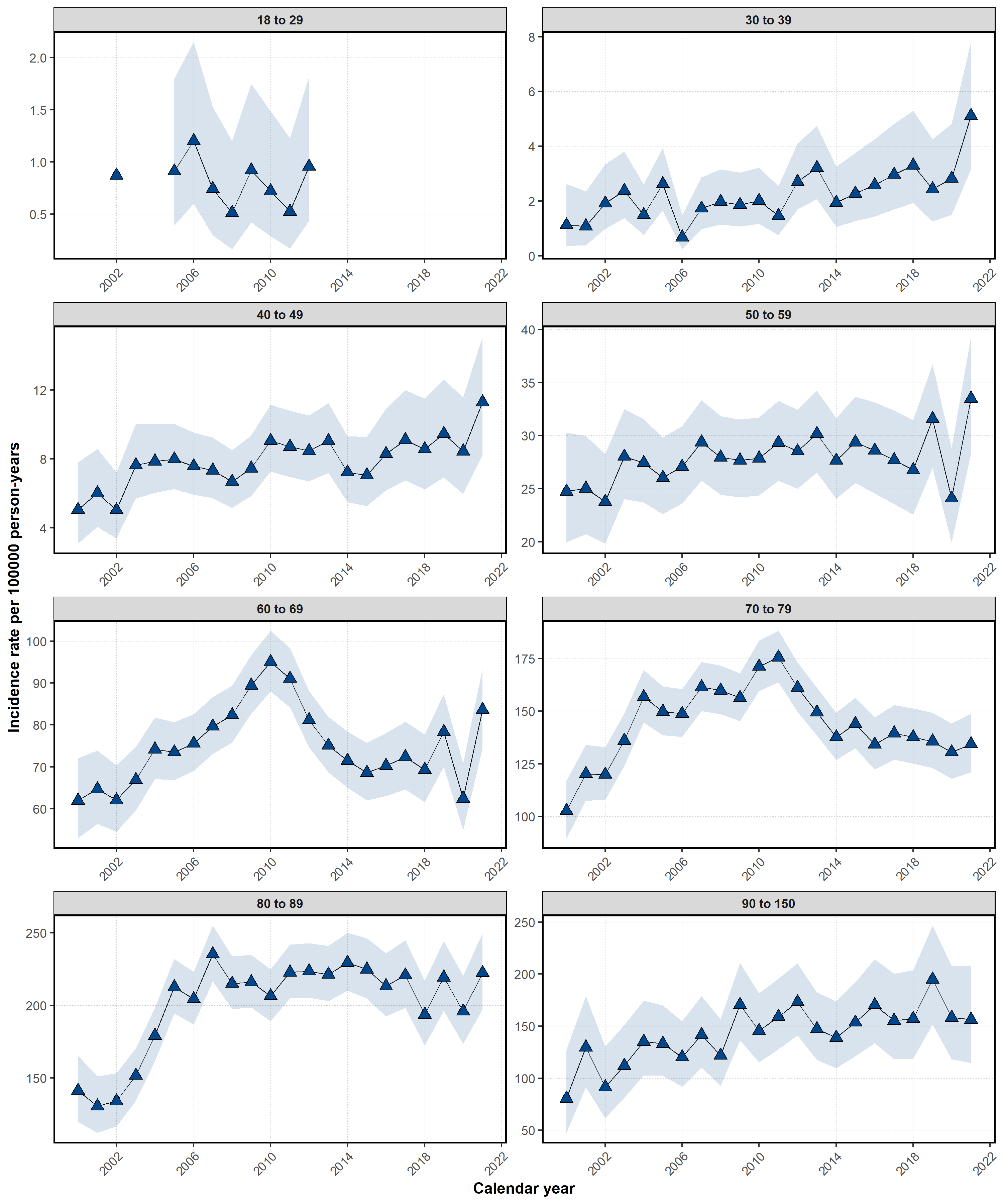


# **S11: Age Standardized annual incidence rates for rectal cancer stratified by age group for CPRD GOLD.**


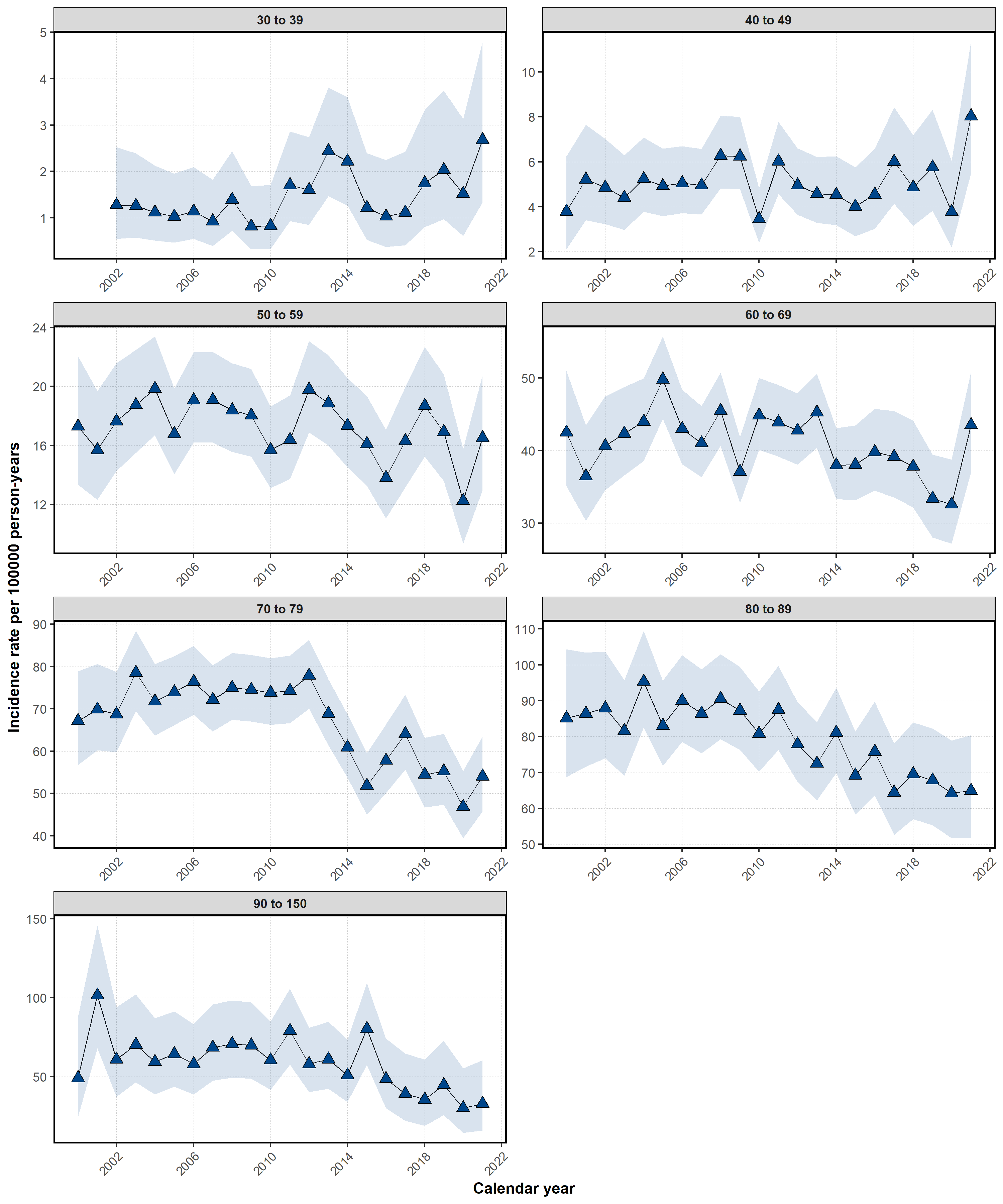


# **S12: Kaplan-Meier survival curve of colorectal cancer by database and sex**





# **S13: Survival (%) after 1, 5 and 10 years after colorectal cancer diagnosis stratified by database and sex.**

| **Time** | **Sex** | **% Survival (95% CI)** | **Database** |
| --- | --- | --- | --- |
| 1 | Male | 79.4 (79.0 - 79.7) | Aurum |
| 5 |  | 50.7 (50.3 - 51.2) |  |
| 10 |  | 37.1 (36.6 - 37.6) |  |
| 1 | Female | 78.2 (77.8 - 78.6) |  |
| 5 |  | 52.9 (52.4 - 53.5) |  |
| 10 |  | 41.2 (40.6 - 41.8) |  |
| 1 | Male | 78.9 (78.4 - 79.4) | GOLD |
| 5 |  | 50.3 (49.7 – 51.0) |  |
| 10 |  | 36.8 (36.1 - 37.5) |  |
| 1 | Female | 77.6 (77.1 - 78.2) |  |
| 5 |  | 52.8 (52.1 - 53.5) |  |
| 10 |  | 40.7 (39.8 - 41.5) |  |

# **S14: Median survival stratified by database and age group.**

| **Age Group** | **Median survival in years (%95 CI)** | **n persons** | **n events** | **Database** |
| --- | --- | --- | --- | --- |
| 18 to 29 | Not achieved | 230 | 76 | Aurum |
| 30 to 39 | Not achieved | 1178 | 346 |  |
| 40 to 49 | Not achieved | 3714 | 1267 |  |
| 50 to 59 | 15.3 (13.8 - 16.4) | 11389 | 4162 |  |
| 60 to 69 | 10.8 (10.4 - 11.3) | 24043 | 9774 |  |
| 70 to 79 | 5.8 (5.6 - 6.0) | 31523 | 16368 |  |
| 80 to 89 | 2.6 (2.5 - 2.7) | 22648 | 14175 |  |
| 90 + | 1.0 (1.0 - 1.1) | 3844 | 2726 |  |
| 18 to 29 | Not achieved | 122 | 29 | GOLD |
| 30 to 39 | Not achieved | 522 | 145 |  |
| 40 to 49 | Not achieved | 1991 | 682 |  |
| 50 to 59 | 15.6 (13.8 - 19.3) | 6322 | 2227 |  |
| 60 to 69 | 10.4 (9.8 - 11.1) | 13375 | 5324 |  |
| 70 to 79 | 5.6 (5.3 - 5.8) | 17083 | 8808 |  |
| 80 to 89 | 2.5 (2.4 - 2.6) | 11860 | 7525 |  |
| 90 + | 1.0 (0.9 - 1.1) | 1823 | 1344 |  |

Not achieved: Median survival was not achieved in study period.

# **S15: One-, five- and ten-year survival (95% confidence intervals) of colorectal cancer stratified by database and age group.**

| **Age Group** | **One-year Survival (%)** | | **Five-year Survival (%)** | | **Ten-year Survival (%)** | |
| --- | --- | --- | --- | --- | --- | --- |
|  | **Aurum** | **GOLD** | **Aurum** | **GOLD** | **Aurum** | **GOLD** |
| 18-29 | 87.2 (82.8 - 91.8) | 89.6 (84.2 - 95.4) | 60.9 (53.9 - 68.8) | 70.8 (61.9 - 80.9) | 54.5 (46.8 - 63.4) | 67.4 (57.2 - 79.5) |
| 30-39 | 86.4 (84.3 - 88.5) | 89.5 (86.8 - 92.3) | 65.4 (62.3 - 68.8) | 67.1 (62.5 – 72.0) | 58.6 (54.9 - 62.5) | 63.0 (58.0 - 68.5) |
| 40-49 | 87.4 (86.3 - 88.5) | 85.5 (83.9 - 87.1) | 63.3 (61.6 - 65.1) | 62.8 (60.5 - 65.3) | 57.7 (55.8 - 59.6) | 57.8 (55.3 - 60.5) |
| 50-59 | 87.3 (86.6 - 87.9) | 86.6 (85.8 - 87.5) | 62.5 (61.5 - 63.5) | 63.7 (62.4 - 65.1) | 55.1 (54.0 - 56.2) | 55.7 (54.2 - 57.3) |
| 60-69 | 85.3 (84.9 - 85.8) | 84.7 (84.0 - 85.3) | 61.9 (61.2 - 62.6) | 61.3 (60.3 - 62.2) | 51.5 (50.7 - 52.2) | 50.7 (49.6 - 51.8) |
| 70-79 | 79.6 (79.2 - 80.1) | 79.2 (78.6 - 79.8) | 52.7 (52.1 - 53.3) | 52.1 (51.3 - 52.9) | 37.2 (36.5 - 37.9) | 36.3 (35.3 - 37.2) |
| 80-89 | 69.2 (68.5 - 69.8) | 68.1 (67.2 - 68.9) | 36.1 (35.4 - 36.8) | 34.4 (33.5 - 35.5) | 17.7 (16.9 - 18.5) | 16.8 (15.8 - 17.9) |
| 90+ | 51 (49.3 - 52.7) | 48.7 (46.4 - 51.2) | 14 (12.6 - 15.5) | 15.1 (13.1 - 17.3) | 3.3 (2.2 - 4.8) | 5.0 (3.4 - 7.4) |

# **S16: Survival after 1 and 5 years stratified by database, calendar year for whole population and sex.**

| **Calendar Year** | **Time (years)** | **% Survival (95% CI)** | **Sex** | **Database** |
| --- | --- | --- | --- | --- |
| 2000 to 2004 | 1 | 79.82 (79.21 - 80.43) | Both | CPRD Aurum |
| 2005 to 2009 | 1 | 77.85 (77.29 - 78.40) |  |  |
| 2010 to 2014 | 1 | 78.80 (78.28 - 79.32) |  |  |
| 2015 to 2019 | 1 | 79.71 (79.21 - 80.21) |  |  |
| 2000 to 2004 | 1 | 76.80 (75.82 - 77.79) |  | CPRD GOLD |
| 2005 to 2009 | 1 | 77.66 (76.96 - 78.36) |  |  |
| 2010 to 2014 | 1 | 79.54 (78.88 - 80.21) |  |  |
| 2015 to 2019 | 1 | 79.30 (78.46 - 80.14) |  |  |
| 2020 to 2021 | 1 | 76.32 (74.57 - 78.12) |  |  |
| 2000 to 2004 | 1 | 79.23 (78.32 - 80.15) | Female | CPRD Aurum |
| 2005 to 2009 | 1 | 77.53 (76.70 - 78.37) |  |  |
| 2010 to 2014 | 1 | 78.25 (77.46 - 79.06) |  |  |
| 2015 to 2019 | 1 | 78.65 (77.88 - 79.42) |  |  |
| 2000 to 2004 | 1 | 76.48 (75.02 - 77.97) |  | CPRD GOLD |
| 2005 to 2009 | 1 | 77.44 (76.40 - 78.50) |  |  |
| 2010 to 2014 | 1 | 78.11 (77.09 - 79.15) |  |  |
| 2015 to 2019 | 1 | 78.29 (77.02 - 79.58) |  |  |
| 2020 to 2021 | 1 | 75.59 (72.94 - 78.32) |  |  |
| 2000 to 2004 | 1 | 80.30 (79.49 - 81.12) | Male | CPRD Aurum |
| 2005 to 2009 | 1 | 78.10 (77.36 - 78.85) |  |  |
| 2010 to 2014 | 1 | 79.22 (78.53 - 79.90) |  |  |
| 2015 to 2019 | 1 | 80.53 (79.88 - 81.19) |  |  |
| 2000 to 2004 | 1 | 77.07 (75.75 - 78.41) |  | CPRD GOLD |
| 2005 to 2009 | 1 | 77.83 (76.90 - 78.77) |  |  |
| 2010 to 2014 | 1 | 80.65 (79.79 - 81.53) |  |  |
| 2015 to 2019 | 1 | 80.10 (79.00 - 81.23) |  |  |
| 2020 to 2021 | 1 | 76.90 (74.57 - 79.29) |  |  |
| 2000 to 2004 | 5 | 52.85 (51.34 - 54.40) | Both | CPRD Aurum |
| 2005 to 2009 | 5 | 49.72 (48.61 - 50.86) |  |  |
| 2010 to 2014 | 5 | 50.84 (48.62 - 53.16) |  |  |
| 2015 to 2019 | 5 | 54.73 (53.64 - 55.84) |  |  |
| 2000 to 2004 | 5 | 50.01 (47.33 - 52.84) |  | CPRD GOLD |
| 2005 to 2009 | 5 | 50.35 (48.83 - 51.92) |  |  |
| 2010 to 2014 | 5 | 53.69 (52.22 - 55.20) |  |  |
| 2015 to 2019 | 5 | 53.40 (51.64 - 55.22) |  |  |
| 2000 to 2004 | 5 | 57.07 (55.36 - 58.83) | Female | CPRD Aurum |
| 2005 to 2009 | 5 | 50.77 (49.11 - 52.49) |  |  |
| 2010 to 2014 | 5 | 49.44 (43.88 - 55.71) |  |  |
| 2015 to 2019 | 5 | 55.74 (54.14 - 57.39) |  |  |
| 2000 to 2004 | 5 | 51.78 (47.88 - 56.00) |  | CPRD GOLD |
| 2005 to 2009 | 5 | 50.61 (48.06 - 53.30) |  |  |
| 2010 to 2014 | 5 | 53.64 (51.44 - 55.93) |  |  |
| 2015 to 2019 | 5 | 55.79 (53.33 - 58.36) |  |  |
| 2000 to 2004 | 5 | 49.49 (47.22 - 51.88) | Male | CPRD Aurum |
| 2005 to 2009 | 5 | 48.86 (47.38 - 50.38) |  |  |
| 2010 to 2014 | 5 | 51.38 (49.82 - 52.98) |  |  |
| 2015 to 2019 | 5 | 53.92 (52.44 - 55.43) |  |  |
| 2000 to 2004 | 5 | 48.73 (45.13 - 52.60) |  | CPRD GOLD |
| 2005 to 2009 | 5 | 50.15 (48.35 - 52.01) |  |  |
| 2010 to 2014 | 5 | 53.73 (51.78 - 55.76) |  |  |
| 2015 to 2019 | 5 | 51.52 (49.07 - 54.09) |  |  |

CI: confidence interval
